# Epidemiological Consequences of Viral Interference: A Mathematical Modeling Study of Two Interacting Viruses

**DOI:** 10.3389/fmicb.2022.830423

**Published:** 2022-03-11

**Authors:** Lubna Pinky, Hana M. Dobrovolny

**Affiliations:** ^1^School of Health Professions, Eastern Virginia Medical School, Norfolk, VA, United States; ^2^Department of Physics & Astronomy, Texas Christian University, Fort Worth, TX, United States

**Keywords:** SARS-CoV-2, influenza, respiratory syncytial virus, rhinovirus, viral interference, mathematical model

## Abstract

Some viruses have the ability to block or suppress growth of other viruses when simultaneously present in the same host. This type of viral interference or viral block has been suggested as a potential interaction between some respiratory viruses including SARS-CoV-2 and other co-circulating respiratory viruses. We explore how one virus' ability to block infection with another within a single host affects spread of the viruses within a susceptible population using a compartmental epidemiological model. We find that population-level effect of viral block is a decrease in the number of people infected with the suppressed virus. This effect is most pronounced when the viruses have similar epidemiological parameters. We use the model to simulate co-circulating epidemics of SARS-CoV-2 and influenza, respiratory syncytial virus (RSV), and rhinovirus, finding that co-circulation of SARS-CoV-2 and RSV causes the most suppression of SARS-CoV-2. Paradoxically, co-circulation of SARS-CoV-2 and influenza or rhinovirus results in almost no change in the SARS-CoV-2 epidemic, but causes a shift in the timing of the influenza and rhinovirus epidemics.

## 1. Introduction

A novel coronavirus first detected in Wuhan, China in late 2019 spread rapidly around the world (Chen et al., 2020; Wu et al., 2020). While many people infected with the virus remain asymptomatic, a significant number of people develop COVID-19 (Dong et al., 2020; Verity et al., 2020)—a disease characterized by severe respiratory distress that leads to hospitalization and potentially death (Goyal et al., 2020; Jiang et al., 2020; Sun et al., 2020). The first waves of SARS-CoV-2 infection occurred in the late spring and summer months when there are few co-circulating respiratory viruses. However, subsequent waves have occurred at times when we normally expect seasonal circulation of viruses such as influenza and respiratory syncytial virus (RSV) (Choe et al., 2019; DeGroote et al., 2020). There was a fear that the combined effect of SARS-CoV-2 and illness due to these other respiratory viruses could lead to an excessive burden on the healthcare system (Kissler et al., 2020; Xu and Li, 2020), but in many parts of the world, the usual seasonal respiratory virus epidemics did not materialize (Casalegno et al., 2021; Eisen et al., 2021; Le Hingrat et al., 2021; Liu et al., 2021; Tempia et al., 2021; Williams et al., 2021). It's not clear if the suppression of other respiratory viruses is due to an interaction between SARS-CoV-2 and these other viruses or due to non-pharmaceutical interventions imposed to control the spread of SARS-CoV-2 (Oh et al., 2021; Redlberger-Fritz et al., 2021; Wagatsuma et al., 2021).

There are observational studies that have noted a lack of SARS-CoV-2 coinfections with other respiratory viruses within a single host (Blasco et al., 2020; Kim et al., 2020; Nowak et al., 2020; Xing et al., 2020; Alhumaid et al., 2021). While this could be attributed to the limited circulation of other respiratory viruses, other studies suggest that the limited number of coinfections might be due to virus-virus interactions within the host (Dee et al., 2021; Stowe et al., 2021). A recent modeling study has suggested that many respiratory viruses actually suppress SARS-CoV-2 infections (Pinky and Dobrovolny, 2020), potentially preventing patients from contracting SARS-CoV-2 while they are infected with another virus

It is unclear what this type of viral interference means for the eventual co-circulation of SARS-CoV-2 with other respiratory viruses. Studies have noted that outbreaks of other respiratory viruses influence each other's timing. For example, several studies found that circulation of the 2009 pandemic influenza virus was delayed in some regions, coinciding with an outbreak of rhinovirus, suggesting that rhinovirus infections delayed the influenza outbreak (Anestad and Nardbo, 2009, 2011; Casalegno et al., 2010a). Studies also noted that the 2009 pandemic influenza outbreak delayed outbreaks of several other respiratory viruses (Casalegno et al., 2010b; Pascalis et al., 2012; Yang et al., 2012; Sun et al., 2014), but it is not just outbreaks of novel viruses that alter outbreaks of circulating viruses. Influenza and RSV have similar seasonality yet they are never seen to peak at the same time, suggesting some kind of interference (Anestad, 1982; Velasco-Hernandez et al., 2015). Interaction between annual seasonal influenza outbreaks and rhinovirus often seems to have an effect on rhinovirus prevalence (Nickbakhsh et al., 2019) and early annual influenza A outbreaks have been observed to shift the timing of RSV, influenza B, and coronavirus outbreaks in the same year (van Asten et al., 2016). More generally, a recent study investigated covariance of a variety of respiratory virus outbreaks finding both negative and positive covariance between a number of viral pairs (Mair et al., 2019).

Epidemiological models have also been developed in an attempt to understand how viruses interact at the population level. Many of these models assume interaction between the viruses based on some form of cross-immunity (Ackleh and Allen, 2005; Allen and Kirupaharan, 2005; Nuno et al., 2005; Saunders, 1981; Andreason, 2018; Garba et al., 2013; Ackleh and Salceanu, 2014; Bhattacharyya et al., 2015; Baguelin and Eggo, 2018; Almaraz and Gomez-Corral, 2019; Amador et al., 2019; Gutierrez-Jara et al., 2019), finding competitive exclusion or at least a diminished presence of one virus depending on the strength and mathematical formulation of the cross-immunity. Other interactions between viruses have also been considered. One epidemiological model examined the interaction of two viruses (influenza and RSV) under the assumption of one virus being competitively stronger than the other, finding that interaction of the two viruses caused emergence of a biennial fluctuation in the size of epidemics (Velasco-Hernandez et al., 2015). A study of different mechanisms of interaction between two influenza strains (H1N1 and H3N2) determined that the interactions only caused observable interference when the attack rates of the strains were high (Ackerman et al., 1990).

In all these models, the cross-protective immunity or other interaction is assumed to be equivalent for both viruses and typically lasts for the duration of the epidemic, whereas interaction through within-host viral interference leads to a one-sided effect that provides only temporary protection against one of the viruses. In this article, we investigate the case of an asymmetric interaction between viruses based on the viral suppression of SARS-CoV-2 by other respiratory viruses. In our model, infection with one virus (influenza, for example) and exposure to the second (SARS-CoV-2) will temporarily prevent infection with the second virus (SARS-CoV-2), but infection with the second virus (SARS-CoV-2) and exposure to the first (influenza) will lead to coinfection. We find that this type of interaction leads to a smaller epidemic of the suppressed virus, but can also alter the timing of the epidemic of the dominant virus.

## 2. Materials and Methods

### 2.1. Mathematical Model

Spread of an infectious disease through a population is often modeled using ordinary differential equation (ODE) compartment models. The common basis for many of these epidemiological models is the Susceptible-Exposed-Infectious-Recovered (SEIR) model where each class of the population is represented by a compartment and movement of people between compartments is governed by ODEs (Hethcote, 2000). We extend the SEIR compartmental model to include two viruses,


(1) $$\begin{array}{l} \text{ Susceptible}:\frac{dS}{dt}=-\frac{\beta_{1}}{N}S(I_{1}+I_{3}+I_{1}^{\left(2\right)})\\ \;-\frac{\beta_{2}}{N}S(I_{2}+I_{3}+E_{3}+I_{2}^{\left(1\right)})\\ \text{Monoinfected Exposed}:\frac{dE_{1}}{dt}=\frac{\beta_{1}}{N}S(I_{1}+I_{3}+I_{1}^{\left(2\right)})-k_{1}E_{1}\\ \;\frac{dE_{2}}{dt}=\frac{\beta_{2}}{N}S(I_{2}+I_{3}+E_{3}+I_{2}^{\left(1\right)})-k_{2}E_{2}\\ \;-\frac{\beta_{1}}{N}E_{2}(I_{1}+I_{3}+I_{1}^{\left(2\right)})\\ \text{ Coinfected Exposed}:\frac{dE_{3}}{dt}=\frac{\beta_{1}}{N}(E_{2}+I_{2})(I_{1}+I_{3}+I_{1}^{\left(2\right)})\\ \;-k_{1}E_{3}\\ \text{ Monoinfected}:\frac{dI_{1}}{dt}=k_{1}E_{1}-\delta_{1}I_{1}\\ \;\frac{dI_{2}}{dt}=k_{2}E_{2}-\delta_{2}I_{2}\\ \;-\frac{\beta_{1}}{N}I_{2}(I_{1}+I_{3}+I_{1}^{\left(2\right)})\\ \text{ Coinfected}:\frac{dI_{3}}{dt}=k_{1}E_{3}-\delta_{3}I_{3} \end{array}$$

$$\begin{array}{l} \text{Recovered Susceptible}:\frac{dS_{1}^{\left(2\right)}}{dt}=\delta_{2}I_{2}-\frac{\beta_{1}}{N}S_{1}^{\left(2\right)}(I_{1}+I_{3}+I_{1}^{\left(2\right)})\\ \;\frac{dS_{2}^{\left(1\right)}}{dt}=\delta_{1}I_{1}\\ \;-\frac{\beta_{2}}{N}S_{2}^{\left(1\right)}(I_{2}+I_{3}+E_{3}+I_{2}^{\left(1\right)})\\ \text{ Recovered Exposed}:\frac{dE_{1}^{\left(2\right)}}{dt}=\frac{\beta_{1}}{N}S_{1}^{\left(2\right)}(I_{1}+I_{3}+I_{1}^{\left(2\right)})-k_{1}E_{1}^{\left(2\right)}\\ \;\frac{dE_{2}^{\left(1\right)}}{dt}=\frac{\beta_{2}}{N}S_{2}^{\left(1\right)}(I_{2}+I_{3}+E_{3}+I_{2}^{\left(1\right)})\\ \;-k_{2}E_{2}^{\left(1\right)}\\ \text{ Recovered Infected}:\frac{dI_{1}^{\left(2\right)}}{dt}=k_{1}E_{1}^{\left(2\right)}-\delta_{1}I_{1}^{\left(2\right)}\\ \;\frac{dI_{2}^{\left(1\right)}}{dt}=k_{2}E_{2}^{\left(1\right)}-\delta_{2}I_{2}^{\left(1\right)}\\ \text{ Recovered}:\frac{dR}{dt}=\delta_{2}I_{2}^{\left(1\right)}+\delta_{1}I_{1}^{\left(2\right)}+\delta_{3}I_{3}. \end{array}$$

**Figure 1:**
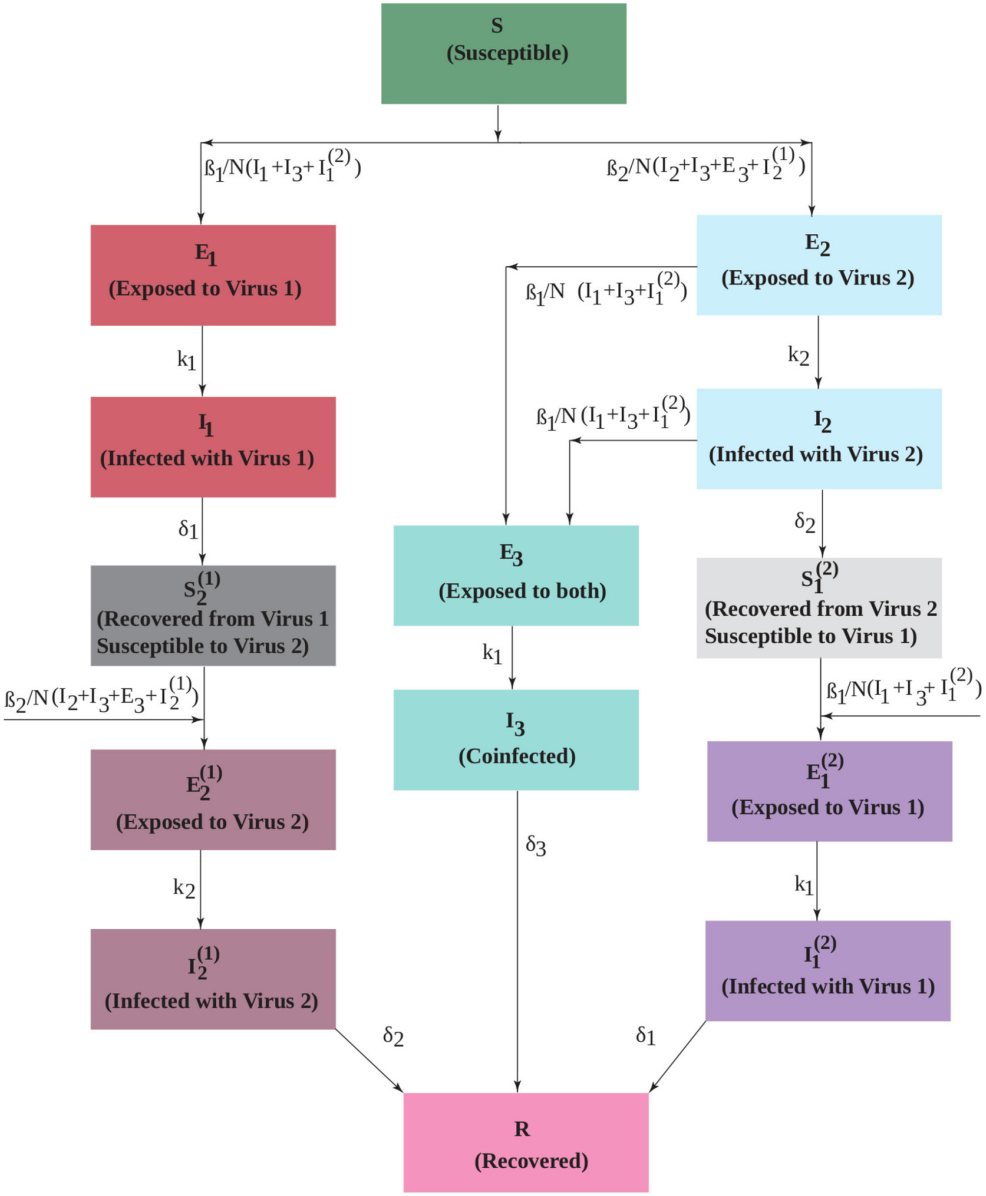
An extended SEIR model that includes two interacting viruses. Infection with virus 1 temporarily blocks infection with virus 2, but infection with virus 2 can lead to coinfection upon exposure to virus 1. The transmission rate of each virus is β_*i*_. The duration of the exposed period is 1/*k*_*i*_ and the duration of the infectious period is 1/δ_*i*_.

The model diagram is given in Figure 1. Initially, the population is susceptible to both viruses (*S*). People can be infected by either virus with force of infection β_*i*_ (*i* = 1, 2) and move into the exposed compartments (*E*_*i*_). Our assumption is that virus 1 (non-SARS-CoV-2 virus such as influenza) blocks infection with virus 2 (SARS-CoV-2) within the host, so people exposed to virus 1 transition to the infectious compartment (*I*_1_). Upon recovery from virus 1, they are immune to virus 1, but are now susceptible to virus 2 ($S_{2}^{\left(1\right)}$). Once infected with the second virus, they move through the exposed ($E_{2}^{\left(1\right)}$) and infectious ($I_{2}^{\left(1\right)}$) stages, after which they are fully recovered (*R*) and immune to both viruses. People who are infected first with virus 2 can develop a coinfection. Thus, people in both the exposed and infected compartments for the second virus (*E*_2_ and *I*_2_) can become coinfected. We assume that coinfected exposed (*E*_3_) people have moved to the infected phase for virus 2, so can infect others with this virus, but are still only exposed to virus 1. After some transition time, they become fully coinfected (*I*_3_) and capable of transmitting both viruses. After some time, coinfected patients also recover and are immune to both viruses. Those patients exposed to virus 2 who do not become coinfected move to the infected compartment (*I*_2_) and recover. At this point, they are immune to virus 2, but are still susceptible to virus 1 ($S_{1}^{\left(2\right)}$). Once infected with virus 1, they move through the corresponding exposed ($E_{1}^{\left(2\right)}$) and infected ($I_{1}^{\left(2\right)}$) compartments to fully recover.

We make a number of simplifying assumptions to keep the model tractable. We assume that the population remains fixed, with total population size *N*, and that births and deaths are negligible over the course of the epidemic. We also do not track deaths due to the infectious diseases. For an epidemic model with a single virus, this would mean that the “recovered” compartment represents all people who are no longer able to participate in the infection, whether it is because they have recovered and are immune or because they have died. In this model, we have compartments with partial recovery where people are immune to one virus, but still susceptible to the other. If someone dies from the first infection, then they cannot be infected with the second virus, so we are over-counting the number of people with sequential infections. This might be substantial, particularly for people infected first with SARS-CoV-2, which has a fairly high death rate (Salzberger et al., 2020). (This assumption is relaxed in the Supplementary Material). We assume that order of infection does not change any of the infection parameters for a particular virus. In this model, these are the force of infection β_*i*_, the incubation period 1/*k*_*i*_, and the recovery rate δ_*i*_ (*i* = 1, 2). We do, however, explore the possibility that coinfection can lead to a longer recovery time.

### 2.2. Simulations

For simulations, we use influenza epidemic parameters (Spencer et al., 2021) as a baseline to investigate interacting viruses (Table 1). We then consider what might happen during a potential next wave of SARS-CoV-2 infection that could coincide with seasonal circulation of other respiratory viruses. We use parameters for influenza, respiratory syncytial virus (RSV), and rhinovirus (RV) derived from fits to several years of seasonal outbreaks in the US (Spencer et al., 2021). Note that, particularly for influenza, this means that we are not modeling any specific strain of influenza. For SARS-CoV-2, we use parameters derived from fits to data from the initial outbreak in China (Anderson et al., 2020; Bentout et al., 2020). Parameters are given in Table 1. This leaves us with one unknown parameter: the coinfection recovery rate δ_3_. Since the recovery rates for SARS-CoV-2 coinfections are unknown, we consider both a faster and slower coinfection recovery rate: δ_3_= 0.1 /d or δ_3_= 0.01 /d. Simulations are run using python's odeint differential equation solver (Bell et al., 2022).

**Table 1:** SARS-CoV-2 (Anderson et al., 2020; Bentout et al., 2020), influenza virus (H1N1) (Spencer et al., 2021), respiratory syncytial virus (RSV) (Spencer et al., 2021), and rhinovirus (RV) (Spencer et al., 2021) parameters for simulation.

**Virus**	**Transmission**	**Exposed**	**Infection**	**Coinfection**
	**rate**	**rate**	**recovery**	**recovery**
	**β_*i*_, d^−1^**	***k*_*i*_, d^−1^**	**rate, δ_*i*_, d^−1^**	**rate, δ_3_, d^−1^**
Flu (H1N1)_*i* = 1_	0.35	0.38	0.21	
RSV_*i* = 1_	0.45	0.22	0.13	0.1 or 0.01
RV_*i* = 1_	0.19	0.42	0.10	
SARS-CoV-2_*i* = 2_	0.41	0.20	0.10	

### 2.3. Sensitivity Analysis

We use the partial rank correlation coefficient (PRCC) to assess which model parameters contribute most to changes in epidemic outcome. We allow parameter values to range between ±10% of their base value and use 1,000 different randomly selected parameter combinations to calculate the PRCC. The partial rank correlation coefficient is close to ±1 if there is a high degree of correlation between the independent and dependent variables, with positive values indicating a positive correlation (both increase or decrease together) and negative values indicating a negative correlation. The output variables examined for PRCC are total number of virus 1 infections, total number of virus 2 infections, and the number of coinfections.

## 3. Results

### 3.1. Epidemics With Identical Viruses

We use a standard compartmental SEIR-type ODE model described by Equation (1). In the model, people infected with virus 1 cannot be infected by virus 2, whereas people infected with virus 2 can be coinfected. The model also allows for sequential infections without any asymmetry, i.e., a person can get the other virus after recovering from their initial infection. Infection is initiated by 100 infected individuals for each of the viruses, *I*_*i*_(0) = 100. We assume an initial susceptible population, *S*(0), of 331,002,651 (the population of the US). All other classes are initially set to zero. We start by examining the interaction of two identical viruses using parameters for influenza infection (Table 1).

**Figure 2:**
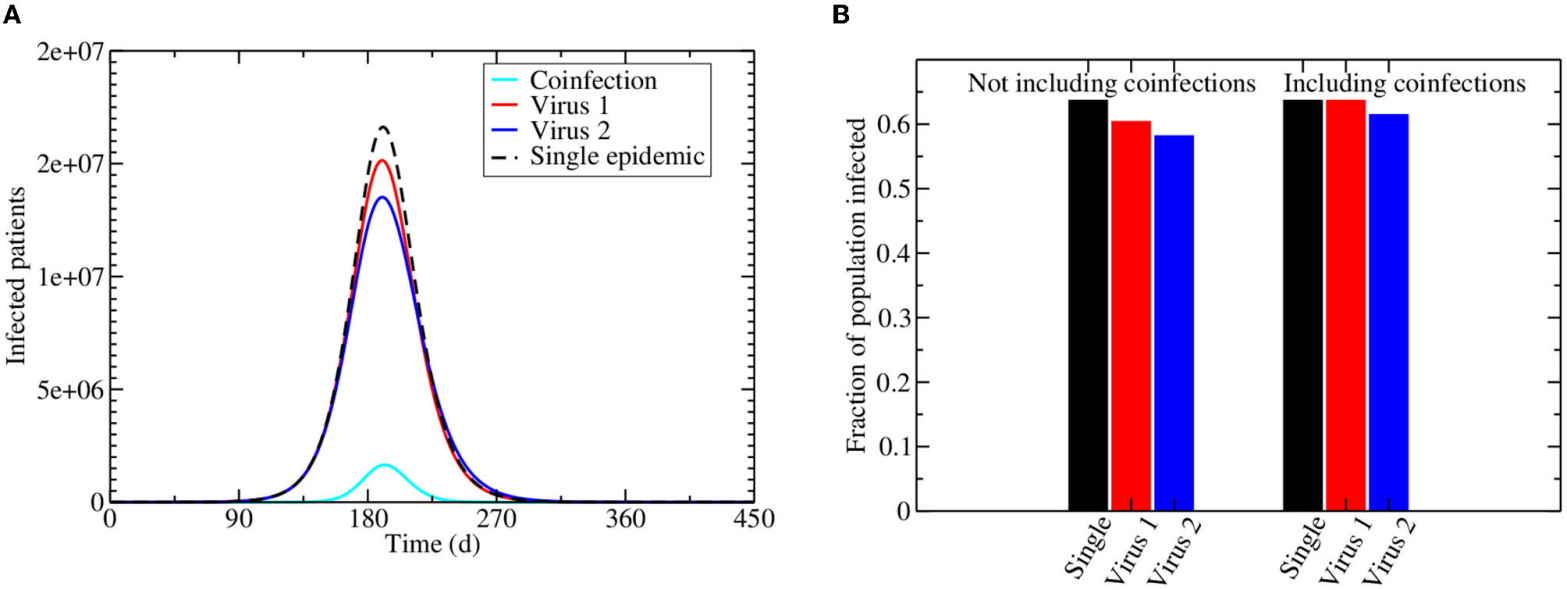
Interacting viral epidemics. Infection is initiated by 100 virus 1 and virus 2 infected individuals assuming the initial number of susceptible population is the current population of the US, i.e., 331,002,651. **(A)** The dashed line shows the trajectory of a single epidemic, while the remaining lines show the number of people infected during a co-circulating epidemic, with red giving the number of people infected with virus 1, blue giving the number of people infected with virus 2, and cyan giving the coinfected population. **(B)** The fraction of the population infected at the end of the epidemic for a single virus (black) or co-circulation of virus 1 (red) and virus 2 (blue). The left bars do not include co-infected people, while the right bars include co-infected people.

Figure 2A shows the projected epidemics caused by two identical viruses circulating in a population, but with virus 1 having the ability to block infection with virus 2. For comparison, the dashed black line shows the epidemic if only one of these viruses is circulating on its own. For both viruses in the co-circulating epidemic, the number of people infected with a single virus (either 1 or 2) is lower than if the virus was circulating without any interaction. The number of people infected with virus 2, however, is more suppressed than the number of people infected with virus 1. Even if we include the coinfected population, there is an overall lowering of the number of people infected with virus 2 in the population, while the total number of people infected with virus 1 stays the same (Figure 2B).

**Figure 3:**
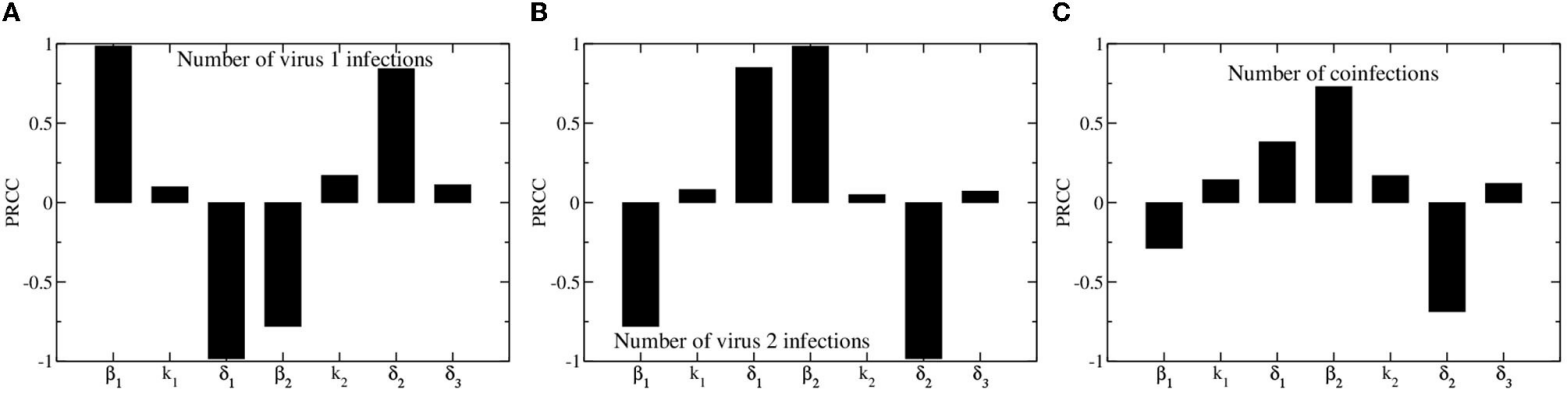
Partial rank correlation coefficients for model parameters. Bars indicate the coefficients, which measure the sensitivity of total number of virus 1 infections **(A)**, virus 2 infections **(B)**, and number of coinfections **(C)** on model parameters.

We use partial rank correlation coefficient (PRCC) to assess how different model parameters affect the outcome of the epidemic. We consider three outcomes: the total number of people infected with virus 1, the total number of people infected with virus 2, and the total number of coinfections. Results of the PRCC are shown in Figure 3. The results for the numbers of people infected with either virus 1 or virus 2 are fairly symmetric, with the total number of people infected with virus 1 depending most strongly on virus 1 infection and recovery rates (β_1_ and δ_1_), and the total number of people infected with virus 2 depending most strongly on virus 2 infection and recovery rates (β_2_ and δ_2_). The asymmetry in the model is most apparent when looking at the parameter dependence of the total number of coinfections. The total number of coinfections depends more strongly on the infection rate and recovery rate of the second virus than on the infection and recovery rates of the first virus. The only way to become coinfected is to first be infected with the second virus, so the more virus 2 infections and the longer they last, the more likely we are to see coinfections.

### 3.2. Epidemics With Different Viruses

In reality the viruses that circulate at the same time are described by different epidemiological parameters. To better understand how differences in viral spread enhance or suppress the population-level interaction of the two viruses, we examine the epidemic when the infection rate (β_1_ or β_2_) of either virus changes. We use three measures of the epidemic curve to assess how the epidemic changes: the peak number of infected, the time of peak, and the cumulative number of infected.

**Figure 4:**
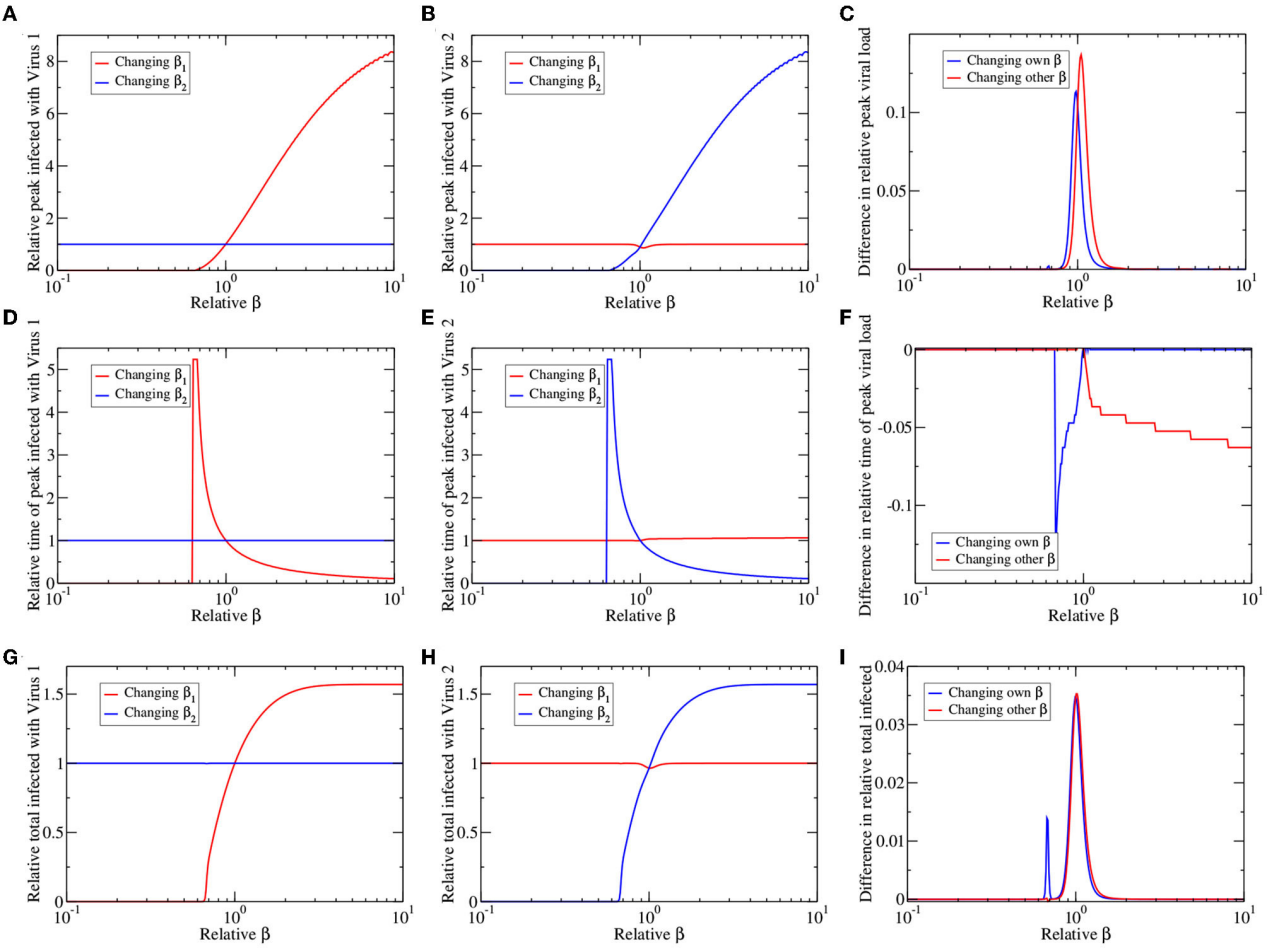
Co-circulating viruses with different infection rates. We change the infection rate of virus 1 (red lines) or virus 2 (blue lines), leaving other parameters the same, and plot the peak number of infected **(A–C)**, time of epidemic peak **(D–F)**, and total number of infected **(G–I)** for virus 1 **(A,D,G)** and virus 2 **(B,E,H)**. All values are presented as relative to their values for a single virus epidemic. **(C,F,I)** The graphs highlight asymmetries in response of the two viruses by subtracting blue lines from corresponding red lines in the left and center columns.

Results of this analysis are shown in Figure 4. The left and center columns show changes in peak number of infected (top row), time of peak (center row), and total infected (bottom row) measured relative to their value in a single virus epidemic. The left column shows results for virus 1 (the dominant virus; Figures 4A,D,G) while the center column shows results for virus 2 (the suppressed virus; Figures 4B,E,H). In each figure we vary either the infection rate of virus one (β_1_) or the infection rate of virus 2 (β_2_). Thus, varying β_1_ increases the peak number of infected for virus 1 (red line; Figure 4A), but has little visible effect on the peak number of infected for virus 2 (red line; Figure 4B). Varying β_2_ has the opposite effect (blue lines; Figures 4A,B). Altogether, we see that varying β_*i*_ causes changes primarily in the epidemic of virus *i*, where there is a minimum β_*i*_ needed for the presence of an epidemic. Beyond this threshold, as β_*i*_ increases, there is a higher peak number of infections (Figures 4A,B), an earlier time of peak (Figures 4D,E) and higher total number of infections (Figures 4G,H). There is little change in the epidemic time course of one virus as the infection rate of the other virus is varied.

There is also very little obvious asymmetry in the graphs; changing the infection rate of the dominant and suppressed virus appears to have more or less the same outcome. However, if we look carefully, there is a small dip in virus 2 response to changes in β_1_ (near relative β = 1) that is not apparent in the response of virus 1 to changes in β_2_ of the left column. To highlight this asymmetry, we subtract the direct responses (virus *i* response to change in β_*i*_) of virus 1 and virus 2, as well as subtract the indirect responses (virus *i* response to change in β_*j*_) of virus 1 and virus 2, with the results given in Figures 4C,F,I. Any asymmetry in the response to changes in infection rate is confined to a small range near equal infection rates when the viruses have similar epidemiological characteristics. The dominant virus has a slightly higher peak number of infections, higher total infections and earlier time of peak than the suppressed virus, no matter which infection rate is varied (either β_1_ or β_2_). Thus, the within host suppression of one virus by another is reflected in decreased population-level spread of the suppressed virus.

### 3.3. SARS-CoV-2 and Other Respiratory Viruses

**Figure 5:**
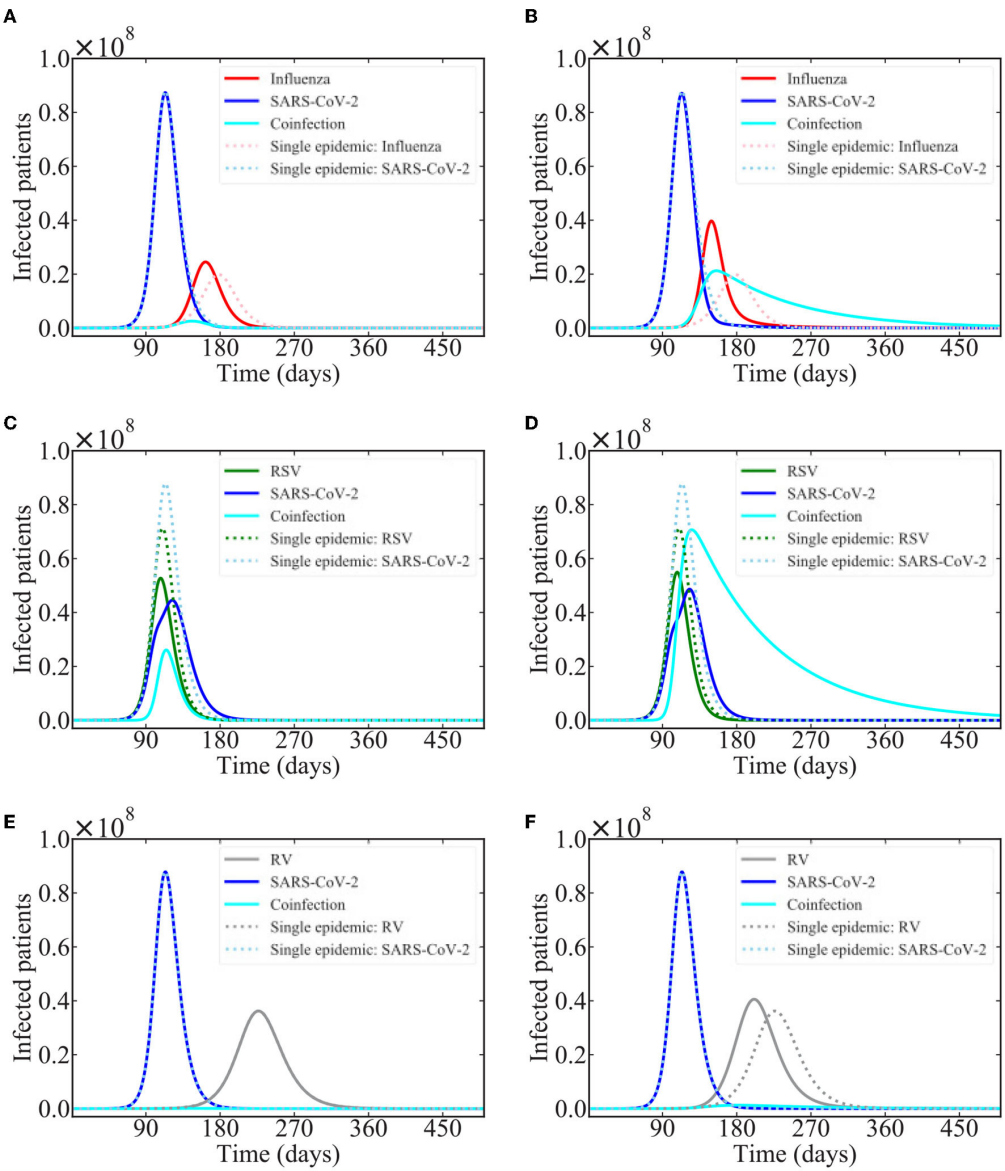
Common respiratory viruses co-circulating with SARS-CoV-2. Graphs show the model predictions for interacting epidemics of SARS-CoV-2 and influenza **(A,B)**, RSV **(C,D)**, and rhinovirus **(E,F)** assuming that SARS-CoV-2 is suppressed during coinfections with each of these viruses. **(A,C,E)** assume a coinfection recovery rate of 0.1 /d while **(B,D,F)** assume a coinfection recovery rate of 0.01 /d.

Based on the results of a recent modeling study that suggests that SARS-CoV-2 is suppressed by several common respiratory viruses (Pinky and Dobrovolny, 2020), we investigated interacting epidemics of SARS-CoV-2 with influenza, respiratory syncytial virus (RSV), and rhinovirus (RV). We start both epidemics at the same time with the same number of infected people (100), which might not be entirely realistic. Model predictions of the epidemics are shown in Figure 5. Note that these simulations use parameters describing spread of each of these viruses in a typical epidemic season without any sort of non-pharmaceutical interventions (NPI) such as mask-wearing, social distancing, or closures/lockdowns. Thus, the predictions are more representative of what could happen in coming years rather than what occurred in 2020 or even in 2021 when some of these NPIs were at least partially in place.

In the case of influenza and rhinovirus, the influenza and RV epidemics are predicted to start largely after SARS-CoV-2 has run its course, so there is little effect of the interaction on the time course of the SARS-CoV-2 epidemic. There is, however, a noticeable effect on the time course of the influenza and RV epidemics, which are shifted, and occur earlier than if either virus was circulating alone. This shift is seen for both values of coinfection recovery rate in the case of influenza, but only for the lower recovery rate in the case of RV. The shift occurs when coinfected individuals transmit the infection for a longer period of time than singly infected individuals. While there are few coinfected individuals, the longer recovery time allows them to infect a few more people, speeding up the epidemic.

In the case of SARS-CoV-2 and RSV interacting epidemics, the epidemics are predicted to peak at nearly the same time, so there is a more visible effect of the within-host viral block. The peak of the SARS-CoV-2 epidemic is reduced and delayed as compared to the single epidemic. Additionally, there is a clear change in the slope of the SARS-CoV-2 epidemic curve during the peak of the RSV epidemic, suggesting that the presence of RSV is preventing some SARS-CoV-2 infections.

### 3.4. Extensions to the Base Model

Two possible extensions of the model are examined in the Supplementary Material, but are found to have a small effect on model predictions. The first is including death of patients, a factor that could be particularly important when considering SARS-CoV-2 and influenza, which have higher case fatality rates than many other respiratory viruses (Zhang and Zhao, 2012; Liu et al., 2020; Fan et al., 2021). Inclusion of death decreases the number of people available for a sequential secondary infection, so there is a reduction in the total number of people infected with either virus (Supplementary Figure 1). We also allowed for virus 1-infected patients to become coinfected with virus 2, assuming that the virus 2 infection would be suppressed enough to be asymptomatic, but would stimulate enough immune response to prevent further virus 2 infections for the remainder of the epidemic (Supplementary Figure 3). This assumption leads to a larger number of coinfected patients and even larger suppression of virus 2 since a subset of virus 2 infected people do not transmit the infection (Supplementary Figure 4).

## 4. Discussion

We explored an epidemic involving two viruses that interact via suppression of one virus if the two viruses occur in the same host. Such viral interference is seen in many viral coinfections (Whitakerdowling and Youngner, 1987; Kumar et al., 2018), and was hypothesized as a possible explanation for an apparent lack of SARS-CoV-2 coinfections with other respiratory viruses (Pinky and Dobrovolny, 2020). Our model predicts that the within host suppression of one virus by another manifests in a decrease in prevalence of the suppressed virus at the population level.

One of the big concerns of public health officials is the potential for a surge of sick patients that might overwhelm hospital capacity (Li et al., 2020; Moghadas et al., 2020). There has been particular concern about co-circulation of SARS-CoV-2 and other respiratory viruses, like influenza, that can also cause surges in hospitalization rates (Bertolani et al., 2018). Should both epidemics peak at the same time, hospitals might not have sufficient resources to treat all those in need. Our simulations indicate that there will be a decrease in the prevalence of the suppressed virus, which helps with the hospital capacity issue, although the effect is small and might not be enough to prevent overwhelming of healthcare systems.

Hospitalization rates will also be affected by the clinical severity of coinfections. If coinfections have a protective effect, there could be less strain on medical resources; if coinfections result in more severe clinical outcomes, hospitals could be even more strained. Some studies have found that coinfections decrease disease severity (Martin et al., 2011, 2013) or are at least no more severe than mono-infections (Brand et al., 2012; Martin et al., 2013; Asner et al., 2014; Rotzen-Ostlund et al., 2014; Mexico Emerging Infect Dis, 2019; Xiang et al., 2021), although others have found that coinfections can be more severe than mono-infections (Waner, 1994; Goka et al., 2015; Alosaimi et al., 2021; Musuuza et al., 2021). In the case of SARS-CoV-2 coinfections, studies are equally mixed about the severity of clinical disease, with some studies indicating a protective effect (Chekuri et al., 2021; Goldberg et al., 2021), others showing a worsening of clinical outcomes (Alosaimi et al., 2021; Stowe et al., 2021), and still others showing no significant difference between SARS-CoV-2 coinfections and mono-infections (Cheng et al., 2021; Guan et al., 2021). A meta-analysis of SARS-CoV-2 coinfections with influenza indicated no overall increase in mortality associated with coinfections, but found that SARS-CoV-2/influenza coinfections had decreased mortality in China and increased mortality in other regions (Guan et al., 2021), suggesting that other factors besides the characteristics of the two pathogens might be involved in determining severity.

Using parameters for influenza, RSV, and rhinovirus, we found that RSV had the most impact on the prevalence of SARS-CoV-2 when the two viruses co-circulate. Of the viruses investigated here, SARS-CoV-2 and RSV have the most similar infection rates (β). According to the analysis of section 3.2, the largest changes occur when the two viruses have similar growth rates, so this is why our model predicts the largest effect for the RSV/SARS-CoV-2 combination. In reality, all of these viruses, and more, are co-circulating together, making it difficult to isolate the interaction of one pair (de Celles et al., 2022). In our simulations, we assume that influenza, RSV, and rhinovirus all suppress SARS-CoV-2, as was suggested in a mathematical modeling study (Pinky and Dobrovolny, 2020). These modeling results have been recently supported by experiments showing suppression of SARS-CoV-2 by rhinovirus in human respiratory epithelium (Dee et al., 2021) and suppression of SARS-CoV-2 viral loads in mice first infected with influenza (Achdout et al., 2021), as well as in ferrets first infected with influenza (Bao et al., 2021). Our model could also be used to simulate the converse, SARS-CoV-2 suppression of other respiratory viruses, although for influenza and rhinovirus, we expect to see little change in the epidemic dynamics since their infection rates are quite different from the infection rate of SARS-CoV-2.

Two extensions of our interaction model, inclusion of patient death and incomplete viral block, are considered in the Supplementary Material, but other factors might also affect the predicted dynamics. For example, asymptomatic infections contribute to spread of influenza (Furuya-Kanamori et al., 2016; Cui et al., 2019), RSV (Munywoki et al., 2015), rhinovirus (Martin et al., 2018), and SARS-CoV-2 (Al-Sadeq and Nasrallah, 2020; Dobrovolny, 2020), but are not explicitly accounted for in our model. Additionally, people infected with SARS-CoV-2 are asked to isolate, keeping them from further spreading the infection (Hellewell et al., 2020). While symptomatic patients with other respiratory viruses will often avoid typical daily activities, isolation measures are not as strict, so this would add an additional asymmetry between the dynamics of SARS-CoV-2 and other respiratory viruses. There is the possibility of other, less direct, interactions between respiratory virus infections. For example, a number of studies have observed a correlation between influenza vaccination and decreased COVID-19 severity in patients (Amato et al., 2020; Marín-Hernández et al., 2021; Zanettini et al., 2020), suggesting a possible interaction between the influenza immune response and SARS-CoV-2 virus. Perhaps the biggest factor not considered in the model is mitigation strategies for reducing spread of SARS-CoV-2, such as wearing of masks and social distancing. These NPIs not only change the time course of the SARS-CoV-2 epidemic, but also help stem the spread of other respiratory viruses (Ngonghala et al., 2020) and are largely thought to be responsible for the lack of typical seasonal respiratory viruses in 2020 (Oh et al., 2021; Redlberger-Fritz et al., 2021; Wagatsuma et al., 2021).

Despite these limitations, our model provides insight into how co-circulating viruses will spread through the population if they participate in viral interference at the within-host level. The model suggests a reduction in the total number of cases of the suppressed virus, although the effect is small, but also suggests a shift in the timing of the peak of the dominant virus is possible.

## Data Availability Statement

The raw data supporting the conclusions of this article will be made available by the authors, without undue reservation.

## Author Contributions

LP and HD: conceptualization, methodology, validation, and writing—review and editing. LP: software and formal analysis. HD: writing—original draft preparation, supervision, and project administration. Both authors have read and agreed to the published version of the manuscript.

## Conflict of Interest

The authors declare that the research was conducted in the absence of any commercial or financial relationships that could be construed as a potential conflict of interest.

## Publisher's Note

All claims expressed in this article are solely those of the authors and do not necessarily represent those of their affiliated organizations, or those of the publisher, the editors and the reviewers. Any product that may be evaluated in this article, or claim that may be made by its manufacturer, is not guaranteed or endorsed by the publisher.
