# Supplementary material for: Epidemiological Consequences of Viral Interference: A Mathematical Modeling Study of Two Interacting Viruses
Source: Front Microbiol. 2022 Mar 11;13:830423. doi: 10.3389/fmicb.2022.830423 (PMC8966706; doi:10.3389/fmicb.2022.830423)
Supplement: Supplementary file 1 [file Data_Sheet_1.PDF]

Supplement for "Epidemiological consequences of viral interference: A mathematical modeling study of two interacting viruses"

Lubna Pinky and Hana M. Dobrovolny

In this supplement we present results from extensions to the model presented in the main manuscript.

1 Including patient deaths

The model in the main text does not include patient death from either virus. This could potentially affect the number of secondary infections predicted by the model since a subset of patients will die after the first infection and are not available for the sequential infection. We explore how this assumption changes model predictions by explicitly accounting for patient death after the first infection. Note that deaths from the second of sequential infections are not explicitly included since people in the final recovered compartment no longer participate in the epidemic.

1.1 Mathematical model

Our model will now allow for patients to enter the final "Recovered" compartment after the first infection, without having to contract both infections (either

sequentially or as coinfections),

$$
\begin{aligned}
\text{Susceptible}: \frac{\mathrm{d}S}{\mathrm{d}t} &= -\frac{\beta_1}{N}S(I_1 + I_3 + I_1^{(2)}) - \frac{\beta_2}{N}S(I_2 + I_3 + E_3 + I_2^{(1)}) \\
\text{Monoinfected Exposed}: \frac{\mathrm{d}E_1}{\mathrm{d}t} &= \frac{\beta_1}{N}S(I_1 + I_3 + I_1^{(2)}) - k_1 E_1 \\
\frac{\mathrm{d}E_2}{\mathrm{d}t} &= \frac{\beta_2}{N}S(I_2 + I_3 + E_3 + I_2^{(1)}) - k_2 E_2 - \frac{\beta_1}{N}E_2(I_1 + I_3 + I_1^{(2)}) \\
\text{Coinfected Exposed}: \frac{\mathrm{d}E_3}{\mathrm{d}t} &= \frac{\beta_1}{N}(E_2 + I_2)(I_1 + I_3 + I_1^{(2)}) - k_1 E_3 \\
\text{Monoinfected}: \frac{\mathrm{d}I_1}{\mathrm{d}t} &= k_1 E_1 - \delta_1 I_1 - \rho_1 \delta_1 I_1 \\
\frac{\mathrm{d}I_2}{\mathrm{d}t} &= k_2 E_2 - \delta_2 I_2 - \rho_2 \delta_2 I_2 - \frac{\beta_1}{N} I_2 (I_1 + I_3 + I_1^{(2)}) \\
\text{Coinfected}: \frac{\mathrm{d}I_3}{\mathrm{d}t} &= k_1 E_3 - \delta_3 I_3 \qquad (1) \\
\text{Recovered Susceptible}: \frac{\mathrm{d}S_1^{(2)}}{\mathrm{d}t} &= \delta_2 I_2 - \frac{\beta_1}{N} S_1^{(2)} (I_1 + I_3 + I_1^{(2)}) \\
\frac{\mathrm{d}S_2^{(1)}}{\mathrm{d}t} &= \delta_1 I_1 - \frac{\beta_2}{N} S_2^{(1)} (I_2 + I_3 + E_3 + I_2^{(1)}) \\
\text{Recovered Exposed}: \frac{\mathrm{d}E_1^{(2)}}{\mathrm{d}t} &= \frac{\beta_1}{N} S_1^{(2)} (I_1 + I_3 + I_1^{(2)}) - k_1 E_1^{(2)} \\
\frac{\mathrm{d}E_2^{(1)}}{\mathrm{d}t} &= \frac{\beta_2}{N} S_2^{(1)} (I_2 + I_3 + E_3 + I_2^{(1)}) - k_2 E_2^{(1)} \\
\text{Recovered Infected}: \frac{\mathrm{d}I_1^{(2)}}{\mathrm{d}t} &= k_1 E_1^{(2)} - \delta_1 I_1^{(2)} \\
\frac{\mathrm{d}I_2^{(1)}}{\mathrm{d}t} &= k_2 E_2^{(1)} - \delta_2 I_2^{(1)} \\
\text{Recovered}: \frac{\mathrm{d}R}{\mathrm{d}t} &= \delta_2 I_2^{(1)} + \delta_1 I_1^{(2)} + \delta_3 I_3 + \rho_1 \delta_1 I_1 + \rho_2 \delta_2 I_2.
\end{aligned}
$$

The parameters ρ_1 and ρ_2 are the proportion of patients who die from virus 1 and virus 2, respectively. We use values of $\rho_1 = 0.0074$ /d for influenza, based on estimates for the 2009 H1N1 pandemic virus [3], and $\rho_2 = 0.0366$ /d for SARS-CoV-2 based on estimates from the second wave of SARS-CoV-2 from many countries [1].

1.2 Results

Fig. 1 shows the results of simulations with the model that explicitly includes death of infected patients under the assumption of identical viruses. The general trend is the same as a model without death, with the peak of both epidemics lower when there is interaction than when each virus circulates independently. However, there is actually a real decrease in the number of people infected with

both viruses in this case, even after accounting for coinfections, since patients who die from infection with virus 1 are removed from the susceptible population of virus 2 and vice versa.

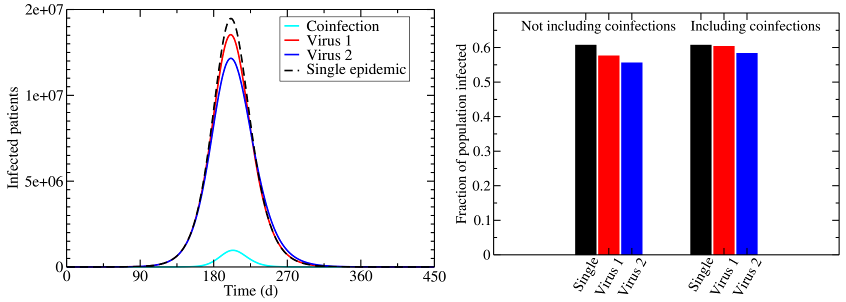

Figure 1: Interacting viral epidemics with death included. (left) The dashed line shows the trajectory of a single epidemic, while the remaining lines show the number of people infected during a co-circulating epidemic, with red giving the number of people infected with virus 1, blue giving the number of people infected with virus 2, and cyan giving the coinfected population. (right) The fraction of the population infected at the end of the epidemic for a single virus (black) or co-circulation of virus 1 (red) and virus 2 (blue). The left bars do not include co-infected people, while the right bars include co-infected people.

We also examined how incorporation of deaths might alter interaction of non-identical viruses. Results of changing the infection rates are shown in Fig. 2. The incorporation of deaths has little effect on the small amount of asymmetry observed in the original model.

2 Incomplete viral block

The within-host simulations of SARS-CoV-2 coinfections with other respiratory viruses suggest that SARS-CoV-2 might not be completely blocked, but might have replication severely suppressed [2]. To include this possibility in our model, we allow for people infected with virus 1 first to become coinfected, but we assume that replication of virus 2 is suppressed enough that they will not be able to transmit the virus.

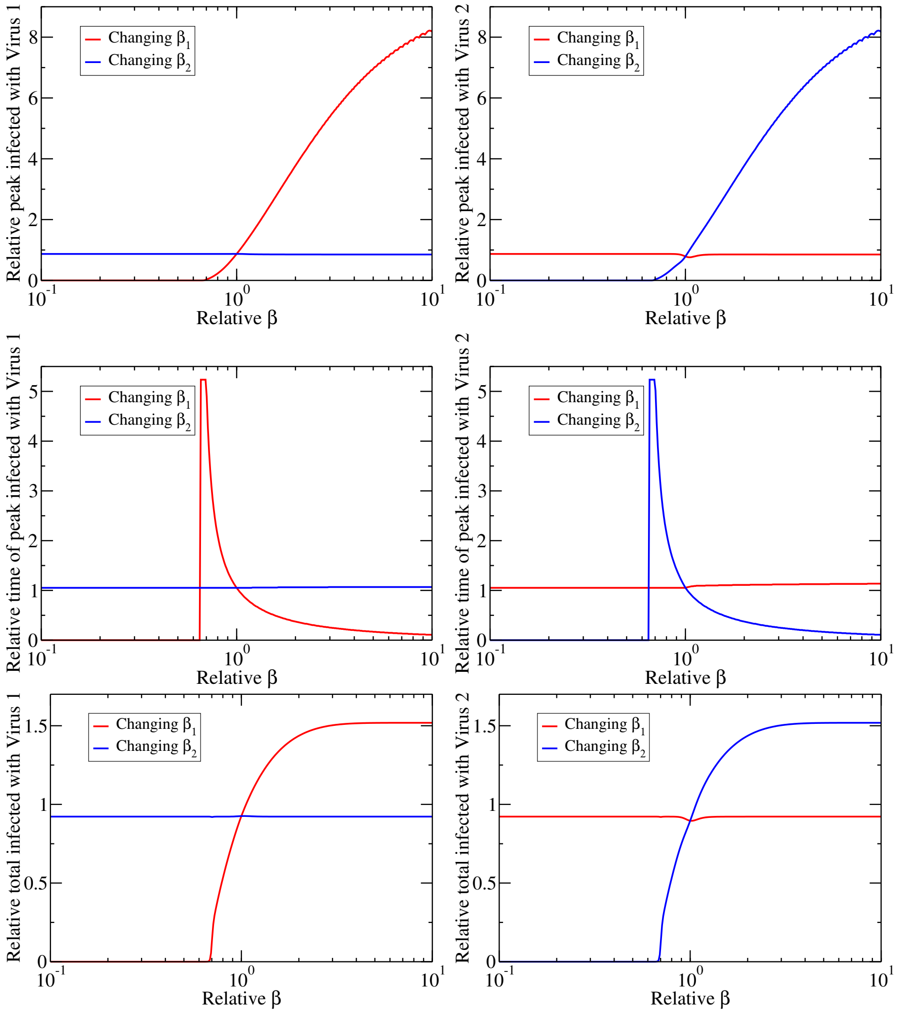

Figure 2: Co-circulating viruses with different infection rates. We change the infection rate of virus 1 (red lines) or virus 2 (blue lines), leaving other parameters the same, and plot the peak number of infected (top row), time of epidemic peak (center row), and total number of infected (bottom row) for virus 1 (left column) and virus 2 (center column). All values are presented as relative to their values for a single virus epidemic.

2.1 Mathematical model

This version of the model allows for coinfection no matter which virus causes the first infection. However, patients infected with virus 1 first cannot transmit virus 2 while coinfected,

$$
\begin{aligned}
\text{Susceptible}: \frac{\mathrm{d}S}{\mathrm{d}t} &= -\frac{\beta_1}{N}S(I_1+I_3+I_1^{(2)}+I_4)-\frac{\beta_2}{N}S(I_2+I_3+E_3+I_2^{(1)})\\
\text{Monoinfected Exposed}: \frac{\mathrm{d}E_1}{\mathrm{d}t} &= \frac{\beta_1}{N}S(I_1+I_3+I_1^{(2)}+I_4)-k_1E_1-\frac{\beta_2}{N}E_1(I_2+I_3+I_2^{(1)})\\
\frac{\mathrm{d}E_2}{\mathrm{d}t} &= \frac{\beta_2}{N}S(I_2+I_3+E_3+I_2^{(1)})-k_2E_2-\frac{\beta_1}{N}E_2(I_1+I_3+I_1^{(2)}+I_4)\\
\text{Coinfected Exposed}: \frac{\mathrm{d}E_3}{\mathrm{d}t} &= \frac{\beta_1}{N}(E_2+I_2)(I_1+I_3+I_1^{(2)}+I_4)-k_1E_3\\
\text{Monoinfected}: \frac{\mathrm{d}I_1}{\mathrm{d}t} &= k_1E_1-\delta_1I_1-\frac{\beta_2}{N}I_1(I_2+I_3+I_2^{(1)})\\
\frac{\mathrm{d}I_2}{\mathrm{d}t} &= k_2E_2-\delta_2I_2-\frac{\beta_1}{N}I_2(I_1+I_3+I_1^{(2)}+I_4)\\
\text{Coinfected}: \frac{\mathrm{d}I_3}{\mathrm{d}t} &= k_1E_3-\delta_3I_3 \qquad (2)\\
\frac{\mathrm{d}I_4}{\mathrm{d}t} &= \frac{\beta_2}{N}E_1(I_2+I_3+I_2^{(1)})-\delta_1I_4\\
\text{Recovered Susceptible}: \frac{\mathrm{d}S_1^{(2)}}{\mathrm{d}t} &= \delta_2I_2-\frac{\beta_1}{N}S_1^{(2)}(I_1+I_3+I_1^{(2)}+I_4)\\
\frac{\mathrm{d}S_2^{(1)}}{\mathrm{d}t} &= \delta_1I_1-\frac{\beta_2}{N}S_2^{(1)}(I_2+I_3+E_3+I_2^{(1)})\\
\text{Recovered Exposed}: \frac{\mathrm{d}E_1^{(2)}}{\mathrm{d}t} &= \frac{\beta_1}{N}S_1^{(2)}(I_1+I_3+I_1^{(2)}+I_4)-k_1E_1^{(2)}\\
\frac{\mathrm{d}E_2^{(1)}}{\mathrm{d}t} &= \frac{\beta_2}{N}S_2^{(1)}(I_2+I_3+E_3+I_2^{(1)})-k_2E_2^{(1)}\\
\text{Recovered Infected}: \frac{\mathrm{d}I_1^{(2)}}{\mathrm{d}t} &= k_1E_1^{(2)}-\delta_1I_1^{(2)}\\
\frac{\mathrm{d}I_2^{(1)}}{\mathrm{d}t} &= k_2E_2^{(1)}-\delta_2I_2^{(1)}\\
\text{Recovered}: \frac{\mathrm{d}R}{\mathrm{d}t} &= \delta_2I_2^{(1)}+\delta_1I_1^{(2)}+\delta_3I_3+\delta_1I_4.
\end{aligned}
$$

The model diagram is given in Fig. 3. Since we are assuming that people initially infected with virus 1 and subsequently coinfected do not transmit or show symptoms of virus 2, they keep the infectious period of virus 1. The only real effect of this coinfection is that they are removed from the virus 2 susceptible population without transmitting virus 2.

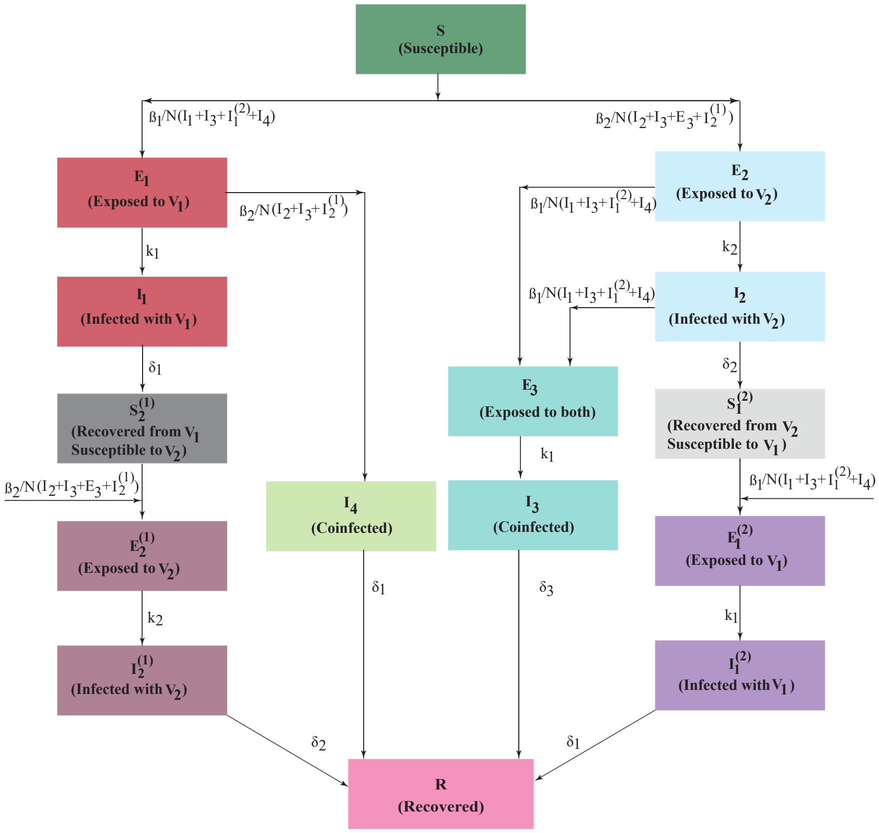

Figure 3: An extended SEIR model that includes two interacting viruses with incomplete viral block. Simultaneous or sequential coinfection with virus 1 and virus 2 are possible with weak blocking of virus 2 by virus 1.

2.2 Results

Fig. 4 shows the results of simulations with the model that allows coinfections no matter which infection is contracted first. There is a higher number of coinfected patients predicted by this model since coinfection can occur no matter which virus infects a person first. We also see that there is less circulation of virus 2 in this model (bar graphs) as compared to when there is full within host suppression of virus 2. In this model people first infected with virus 1 can become coinfected, but cannot transmit virus 2. In the original model, without this route to coinfection, patients would recover from virus 1 and subsequently contract and transmit virus 2. Thus, somewhat paradoxically, partial suppression of virus 2 at the within host level actually leads to more suppression of virus 2 at the population level.

We also examined how partial suppression might alter interaction of non-

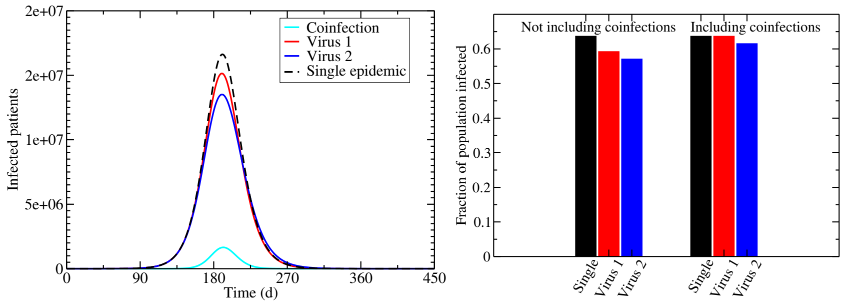

Figure 4: Interacting viral epidemics with only partial suppression of virus 2. (left) The dashed line shows the trajectory of a single epidemic, while the remaining lines show the number of people infected during a co-circulating epidemic, with red giving the number of people infected with virus 1, blue giving the number of people infected with virus 2, and cyan giving the coinfected population. (right) The fraction of the population infected at the end of the epidemic for a single virus (black) or co-circulation of virus 1 (red) and virus 2 (blue). The left bars do not include co-infected people, while the right bars include co-infected people.

identical viruses. Results of changing the infection rates are shown in Fig. 5. The addition of possible coinfections no matter which virus infects someone first also shows few changes from the original model when we consider changing infection rates.

References

[1] Guihong Fan, Zhichun Yang, Qianying Lin, Shi Zhao, Lin Yang, and Daihai He. Decreased case fatality rate of COVID-19 in the second wave: A study in 53 countries or regions. *Transbound. Emerg, Dis.*, September 2020. doi: 10.1111/tbed.13819.

[2] Lubna Pinky and Hana M. Dobrovolny. SARS-CoV-2 coinfections: Could influenza and the common cold be beneficial? *J. Med. Virol.*, pages 1–8, 2020. doi: 10.1016/j.jtbi.2019.01.011.

[3] Jessica Y. Wong, Heath Kelly, Dennis K.M. Ip, Joseph T. Wu, Gabriel M. Leung, and Benjamin J. Cowling. Case fatality risk of influenza A (H1N1pdm09) a systematic review. *Epidemiol.*, 24(6):830–841, November 2013. doi: 10.1097/EDE.0b013e3182a67448.

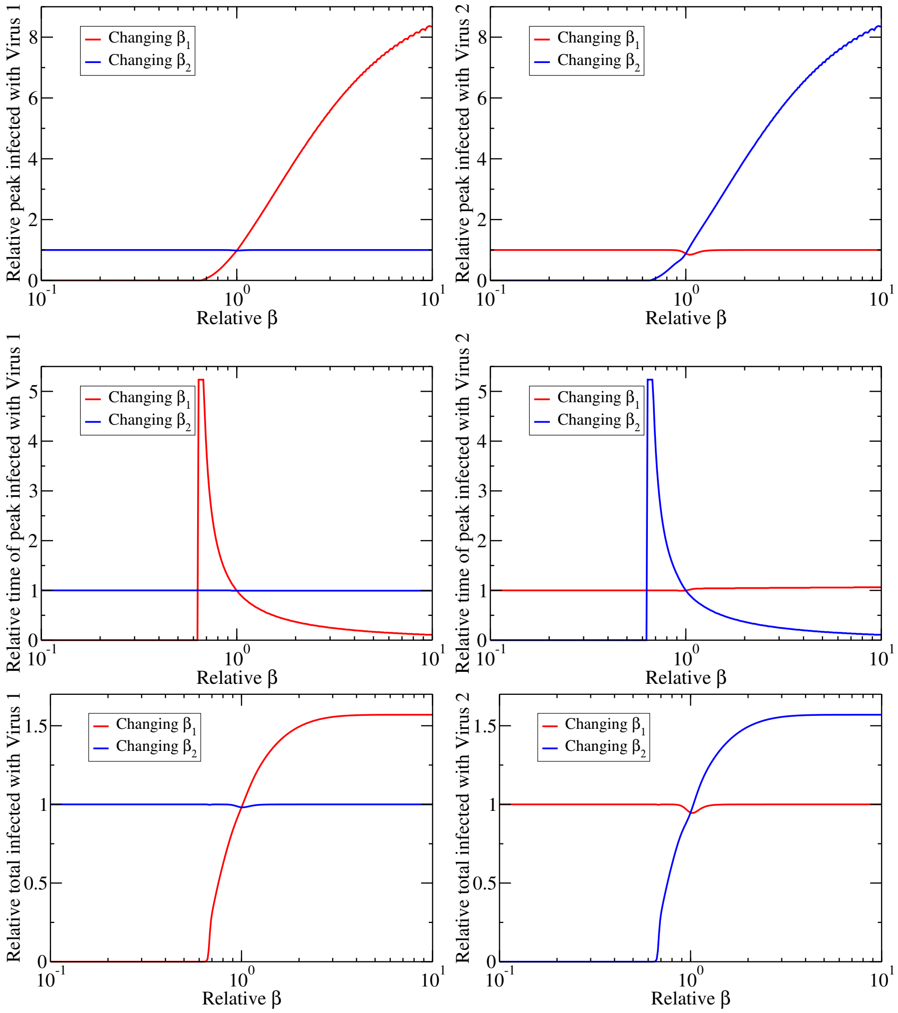

Figure 5: Co-circulating viruses with different infection rates. We change the infection rate of virus 1 (red lines) or virus 2 (blue lines), leaving other parameters the same, and plot the peak number of infected (top row), time of epidemic peak (center row), and total number of infected (bottom row) for virus 1 (left column) and virus 2 (center column). All values are presented as relative to their values for a single virus epidemic.
